# From Spreadsheet To Prediction Tool: A Practical Artificial Intelligence Guide For Urologists

**DOI:** 10.7759/cureus.108082

**Published:** 2026-05-01

**Authors:** Ankit Joshi, Kinju Adhikari, Ravi Taori, Deepak Krishnappa, Aadhar Jain, Lingesh Chelliah, Layeeq Fatima, Raghunath Krishnappa

**Affiliations:** 1 Uro-Oncology and Robotic Surgery, HCG Cancer Centre, Bengaluru, IND

**Keywords:** ai and machine learning, artificial intelligence and medicine, a urologist perspective, data and analytics, predictive model, surgical research, urology education

## Abstract

Artificial intelligence (AI) and machine learning (ML) are transforming urological practice; however, most clinicians lack the technical background required to develop, evaluate, or critically appraise predictive models. Existing resources are often written by data scientists for a technical audience, highlighting the need for a practical, clinician-oriented framework that enables urologists to build and deploy meaningful AI tools using data they already possess. Following an overview of the AI landscape in urology, we present a structured nine-part framework that includes clinical data appraisal; data cleaning and variable engineering; model selection (e.g., logistic regression, Random Forest, Extreme Gradient Boosting (XGBoost), and Cox regression); train-test splitting; cross-validation; performance evaluation (including area under the curve (AUC), calibration, and decision curve analysis); AI-assisted coding using platforms such as Google Colab and large language models (LLMs); web application deployment (e.g., Hugging Face, Gradio, GitHub, Render, and Google Cloud); manuscript preparation aligned with Transparent Reporting of a Multivariable Prediction Model for Individual Prognosis or Diagnosis-Artificial Intelligence (TRIPOD-AI) reporting standards; and ethical considerations for responsible AI deployment. Each component is illustrated with real-world examples and supported by validated prompt templates. Applying this framework to a high-risk prostate cancer cohort, the lead author, without prior programming experience, successfully developed and publicly deployed a validated multi-outcome prediction tool within 72 hours using entirely free, open-source infrastructure. AI-based clinical prediction tools are increasingly accessible to urologists with structured datasets and a systematic approach. This guide aims to democratize AI model development by enabling clinicians to extract actionable insights from existing data, build validated tools, and contribute meaningfully to the evolving landscape of AI-driven urological care, without the need to write code from scratch.

## Introduction

The artificial intelligence moment in urology: why now, why you

In 2016, a deep learning algorithm outperformed dermatologists in identifying skin cancers from clinical photographs [1]. In 2020, an artificial intelligence (AI) model predicted protein structures that had eluded biochemists for decades. In 2023, large language models (LLMs) demonstrated the ability to pass medical licensing examinations. Each of these milestones was initially met with skepticism, yet rapidly became reality. Urology is no exception. AI models have demonstrated non-inferior performance compared to expert radiologists in prostate MRI interpretation [2-4], predicted biochemical recurrence after radical prostatectomy with greater accuracy than established nomograms [5], and stratified patients undergoing androgen deprivation therapy with survival predictions comparable to conventional Cox regression models [6]. Between 2021 and 2024, publications on machine learning (ML) in prostate cancer increased at an average annual rate of 25.4%, with 82% of cumulative output concentrated within this four-year period [7], reflecting a field that has rapidly evolved from theoretical exploration to early clinical integration [8].

Prostate cancer incidence is projected to double by 2040 [9], and the potential implementation of MRI-based population screening is gaining momentum [10]. Both trends are expected to significantly increase the demand for accurate and efficient risk stratification tools. High inter-reader variability in MRI reporting and the relatively low positive predictive value of current diagnostic pathways further support the role of AI-assisted decision-making [3]. The question facing urologists today is not whether AI will change clinical practice; it will. Rather, the key question is whether clinicians will remain passive users of externally developed tools or become active contributors who shape how these tools are designed, which outcomes they predict, and which patient populations they serve.

The problem with how AI is currently taught

The vast majority of published AI methodology guides are written by computer scientists and data engineers. They often assume fluency in Python (Python Software Foundation, Fredericksburg, VA, USA), familiarity with statistical learning theory, and access to advanced computational infrastructure. For clinicians trained in anatomy, pathology, and operative technique, such guides can feel inaccessible, almost like a foreign language. Yet, it is the urologist who understands the clinical context: what a prostate-specific antigen (PSA) trajectory means in a patient treated with enzalutamide, which outcomes truly matter to patients, which variables are reliably recorded, and which clinical questions remain unanswered. This domain knowledge is the most valuable component of any clinically meaningful AI model. Despite this, clinicians are often excluded from model development due to technical barriers that, by 2026, are increasingly surmountable. A key limitation of AI prediction models is their reduced ability to generalize to populations beyond the training dataset. This limitation is well documented in the literature and underscores the importance of external validation prior to clinical deployment [11].

Companion study as proof of concept

All principles outlined in this guide were applied to develop a multi-outcome clinical prediction tool for high-risk prostate cancer patients undergoing neoadjuvant hormonal therapy. The resulting web-based application is freely accessible at https://huggingface.co/spaces/drankitjoshi/naht-predictor [12], demonstrating that the entire pipeline, from data processing to model deployment, can be completed without prior programming experience.

## Technical report

**Figure 1:**
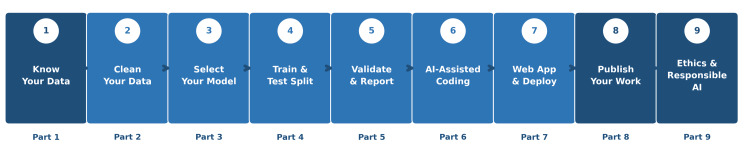
The nine-stage AI model development pipeline for urologists, from raw clinical data to a deployed, published prediction tool. Image Credit: This image was created by the authors using Microsoft PowerPoint (Microsoft Corp., Redmond, WA, USA).

By following this nine-part framework (Figure 1), you will be able to appraise your existing clinical dataset and identify outcomes suitable for prediction modeling; clean and structure raw clinical data; select an appropriate model for your research question; build and validate a prediction model using Google Colab (a free, browser-based platform requiring no installation; Google, Mountain View, CA, USA); use LLMs as coding assistants with tested prompt templates; evaluate model performance using accepted metrics; deploy your model as a publicly accessible web application at no cost; and prepare a manuscript aligned with Transparent Reporting of a Multivariable Prediction Model for Individual Prognosis or Diagnosis-Artificial Intelligence (TRIPOD-AI) reporting standards [13].

Part 1: Know your data

You Probably Already Have What You Need

A common misconception is that building a prediction model requires a large, specially curated research dataset. In reality, many clinically meaningful AI tools are developed using data that already exist within departmental databases, hospital information systems, operative logs, multidisciplinary team (MDT) records, and tumor registries. A single-center retrospective cohort of approximately 150-300 patients, with consistently recorded outcomes and predictor variables, can be sufficient for initial model development, as demonstrated by several published urological AI studies [14-16].

Questions to assess your data readiness

Before beginning model development, four questions help assess whether your dataset is ready. First, do you have at least 100 patients with a defined treatment or disease? Second, do you have at least one clearly measurable outcome such as recurrence, a complication, or a treatment response? Third, have you recorded relevant clinical variables before and/or after treatment? Fourth, is your outcome reliably and consistently defined across all patients? If you can answer yes to all four, you have sufficient data to proceed.

Identifying Your Outcomes

This helps urologists identify the type of outcome they aim to predict and select the most appropriate AI model for that purpose (Table 1).

**Table 1:** Identifying your outcomes. BCR: biochemical recurrence, pCR: pathological complete response.

Outcome type	Urological examples	Model needed
Binary (yes/no)	BCR, positive margins, pCR, 30-day readmission, node positivity	Logistic regression, Random Forest, XGBoost
Time-to-event	Time to BCR, metastasis-free survival, cancer-specific survival	Cox proportional hazards, Kaplan-Meier
Continuous	PSA reduction %, prostate volume change, operative time	Linear regression
Multi-class	Gleason grade group, response category, lymph node burden	Multinomial regression (advanced)

Part 2: Clean your data

Why Data Cleaning Takes Most of Your Time

In most published AI studies, model training and validation are described in detail; however, the substantial time required for data cleaning prior to model development is often underreported. In practice, data preparation accounts for approximately 70%-80% of the total project time [17]. Data cleaning should not be viewed as a limitation of the dataset, but rather as a necessary step, reflecting that clinical data are primarily collected for patient care rather than research. Transforming such data into a research-grade dataset requires deliberate and systematic effort.

Using Google Colab

Google Colab (colab.research.google.com) is a free, browser-based Python environment that requires no installation, dedicated IT support, or specialized computing infrastructure. It operates entirely within a web browser, stores files in Google Drive, and provides sufficient computational capacity for datasets comprising several thousand patients. To begin, navigate to colab.research.google.com and sign in with a Google account. Create a new notebook by selecting “New notebook,” and then upload your dataset via File → Upload to session storage. For efficient workflow, open an AI coding assistant (e.g., Claude (Anthropic, San Francisco, CA, USA) or ChatGPT (OpenAI, San Francisco, CA, USA)) in a separate browser tab.

The following are examples of key prompt templates for data cleaning.

EXAMPLE PROMPT I have a clinical dataset of [N] patients stored as an Excel file called [filename.xlsx]. Columns include: [paste column names and types]. I want to: 1) Load this into Python using pandas, 2) Show the first 5 rows, 3) Count missing values per column, 4) Show the data type of each column. Write clean Python code for Google Colab with comments explaining each step.

EXAMPLE PROMPT I have missing values. For continuous variables (PSA, Age, Volume), impute using median. For binary variables (BCR, Margin_positive), impute using mode. Use sklearn SimpleImputer and show how many values were imputed per column.

Common Data Problems and Solutions

The common data quality problems found in clinical datasets are provided in Table 2.

**Table 2:** Common data quality problems found in clinical datasets, with a real example and a Python solution to fix them.

Problem	Example	Solution
Inconsistent date formats	"01/03/2022" vs "2022-03-01"	Use pd.to_datetime() with errors='coerce'
Text in numeric columns	"N/A", "unknown", "<0.1" in PSA column	Replace with NaN, then impute
Duplicate patients	Same patient twice with different outcomes	df.duplicated() then resolve clinically
Ordinal data as text	T-stage as "T3a", "T3b", "T4"	Map to integers with a dictionary
Outliers	PSA = 9999 (data entry error)	IQR or domain knowledge to cap/exclude
Inconsistent case	"Yes", "yes", "YES" in same column	df[col].str.upper().strip()

Creating Derived Variables

Derived variables are new features created from existing data and often capture clinically meaningful concepts that are not directly recorded. For example, from raw pre- and post-treatment PSA and prostate volume values, one can derive percentage PSA reduction, percentage volume reduction, a binary indicator of PSA normalization, and a T-stage downstaging variable. Each of these may serve as an important predictor in subsequent models.

Part 3: Select your model

**Figure 2:**
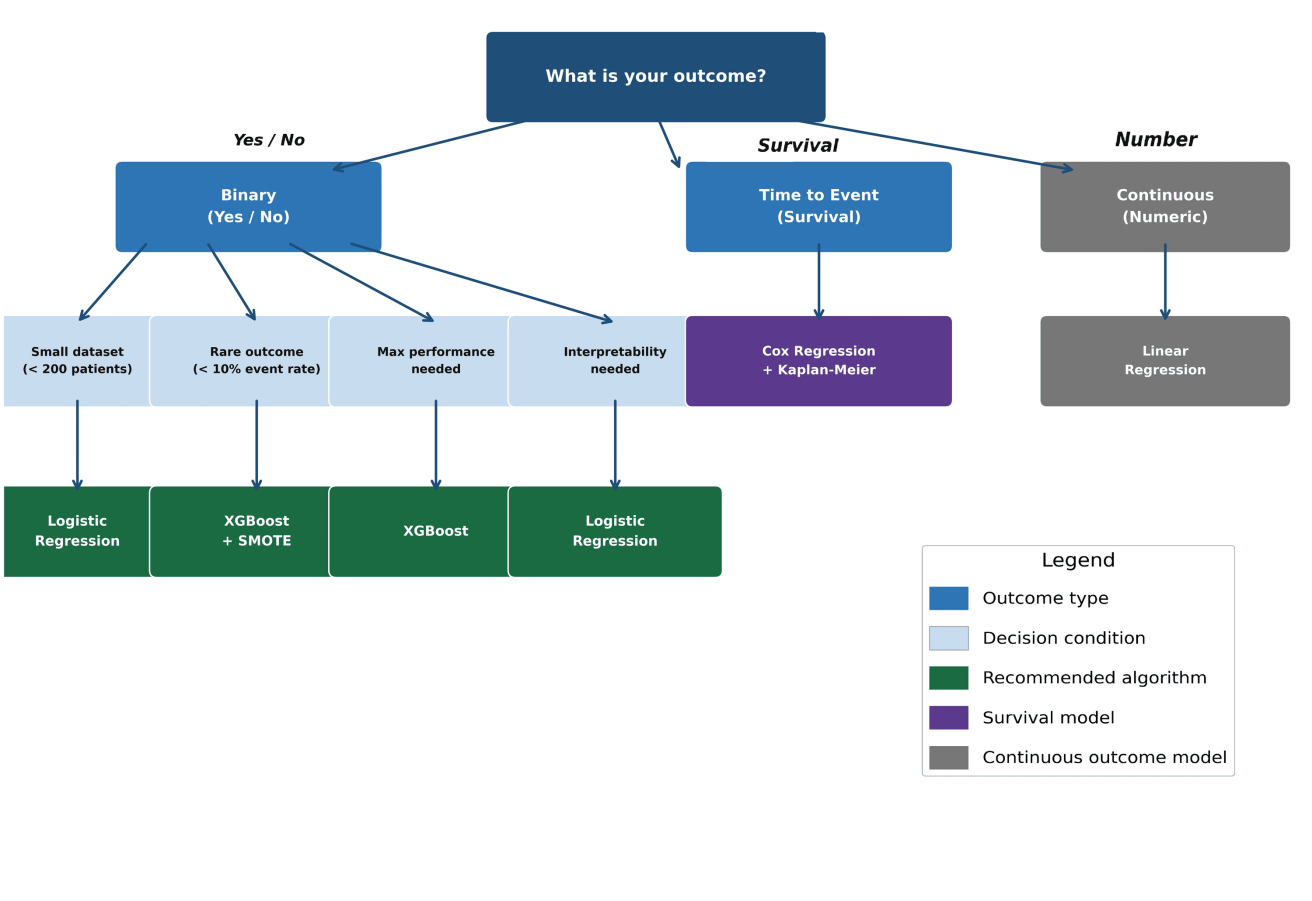
Model selection decision guide for urological prediction studies. SMOTE: Synthetic Minority Over-sampling Technique. Image Credit: This image was created by the authors using Microsoft PowerPoint (Microsoft Corp., Redmond, WA, USA).

The four models described in this section cover the majority of practical urological prediction applications. Figure 2 provides a visual decision guide that maps common clinical questions to the appropriate algorithm and can be used as a quick reference when planning your analysis.

Logistic Regression: The Clinical Workhorse

Logistic regression estimates the probability of a binary outcome as a function of one or more predictor variables and produces odds ratios that are familiar to clinical researchers. It is the most interpretable model discussed in this section. When the dataset is small, the outcome is binary, or maximum interpretability is required for a clinical audience, logistic regression should be considered the first-line approach. It has also formed the basis of widely used nomograms, such as the Partin tables and Memorial Sloan Kettering preoperative models, which have guided urological decision-making for decades [18].

Random Forest: When Relationships Are Complex

Random Forest builds multiple decision trees on random subsets of data and aggregates their predictions. It handles non-linear relationships, variable interactions, and missing data effectively, and provides feature importance scores that identify the most influential predictors. In practice, for a given dataset and outcome, one algorithm will often outperform the others, illustrating that no single method is universally superior across all clinical contexts. Therefore, it is generally recommended to test multiple models and select the one with the best performance on the available data.

Extreme Gradient Boosting (XGBoost): When You Want Maximum Performance

XGBoost builds decision trees sequentially, with each tree learning from the errors of the previous ones. This boosting approach makes it one of the most powerful algorithms for structured tabular clinical data [19]. It is particularly effective when class imbalance is addressed using the Synthetic Minority Over-sampling Technique (SMOTE), which generates synthetic samples of the minority class in the training set to correct imbalance prior to model training [20]. In practice, XGBoost combined with SMOTE can achieve excellent discrimination even for rare outcomes, with event rates as low as 3%-5%, performance levels that are typically difficult to achieve with standard logistic regression in highly imbalanced datasets.

Cox Proportional Hazards: When Timing Matters

Cox regression models the time to an event and accounts for patients lost to follow-up (censored observations). It produces hazard ratios and can be used to generate Kaplan-Meier survival curves stratified by predicted risk groups. ML-based variants, including random survival forests, have demonstrated superior performance to classical Cox regression in some prostate cancer applications [6]. However, the traditional Cox model remains the clinical standard and is often preferred for initial publications due to its familiarity among reviewers and readers.

Part 4: Train-test split

The Train-Test Split

**Figure 3:**
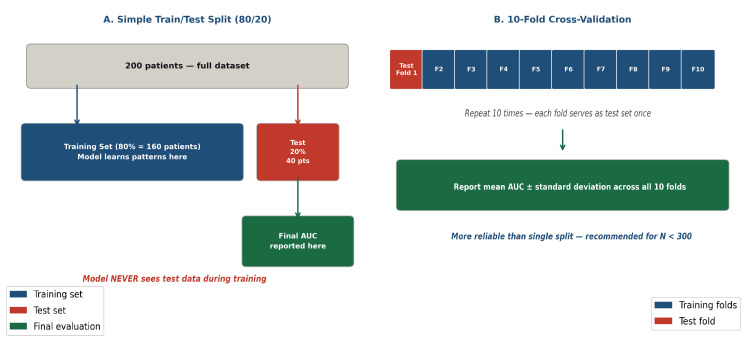
Schematic representation of (A) simple 80/20 train-test split and (B) 10-fold cross-validation. Cross-validation is preferred for smaller datasets to ensure robust performance estimation. Image Credit: This image was created by the authors using Microsoft PowerPoint (Microsoft Corp., Redmond, WA, USA).

If a model is tested on the same data on which it was trained, it may appear to perform exceptionally well, not because it has learned genuine patterns, but because it has effectively memorized the data. This phenomenon, known as overfitting, is one of the most common pitfalls in clinical AI research [11]. To address this, the dataset should be divided into a training set (typically 70%-80%), on which the model is developed, and a test set (20%-30%), which is kept completely separate and used only for final evaluation. This split is schematically illustrated in Figure 3A.

Cross-validation

When a dataset is small (<300 to 400 patients), a single 80/20 split may not provide reliable estimates in either the training or test set. In k-fold cross-validation (typically k = 10), the dataset is divided into k equal parts. The model is trained on k − 1 folds and tested on the remaining fold, a process repeated k times. The final performance is reported as the average across all folds. Cross-validation provides a more stable estimate of model performance but does not replace a held-out test set, as it is an internal validation method. Ideally, published models should include both internal cross-validation and an independent external validation cohort from a different institution [13,21] (Figure 3B).

Part 5: Validate and report

Performance Metrics

**Figure 4:**
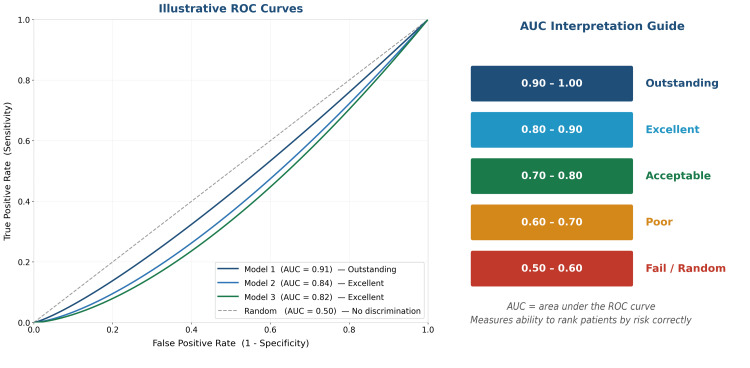
Illustrative ROC curves for three hypothetical prediction models (left). AUC interpretation reference guide for clinical prediction studies (right). AUC: area under the curve, ROC: receiver operating characteristic. Image Credit: This image was created by the authors using Microsoft PowerPoint (Microsoft Corp., Redmond, WA, USA).

Reporting area under the curve (AUC) alone is no longer sufficient for publication. Modern journals and TRIPOD-AI guidelines require a more comprehensive set of performance metrics. Figure 4 provides a visual reference for receiver operating characteristic (ROC) curves and AUC interpretation, while Table 3 defines each metric and its appropriate context.

**Table 3:** Performance metrics for clinical AI prediction models. AI: artificial intelligence, AUC: area under the curve, pCR: pathological complete response.

Metric	What it measures	Target value	When to use
AUC	Overall discrimination: ability to rank patients by risk. 0.5 = random, 1.0 = perfect	>0.80 = good; >0.90 = excellent	All binary models: always report
Sensitivity	Of all patients with the outcome, what % did the model identify?	Context-dependent	When missing a positive case (false negative) is costly
Specificity	Of patients without the outcome, what % did the model correctly classify?	Context-dependent	When false positives carry significant cost
F1 score	Harmonic mean of sensitivity and positive predictive value. Balances false positives and false negatives in a single number	>0.70 = acceptable; higher is better	Essential for rare or imbalanced outcomes (e.g., pCR, node positivity). Always report alongside AUC when the event rate is below 20%
Calibration	Does predicted probability match observed event rate? A model predicting 30% risk should be right ~30% of the time	Calibration slope near 1.0	Essential for clinical use: often neglected
C-index	Survival analysis equivalent of AUC: ranks patients by predicted time-to-event	>0.70 = acceptable	Cox models only
Brier score	Combined calibration and discrimination for probability predictions	Lower is better (<0.25)	Supplement to AUC

TRIPOD-AI Compliance

TRIPOD-AI is an international consensus reporting standard for clinical AI/ML prediction models, published in the BMJ in April 2024 [13]. It supersedes the 2015 TRIPOD statement and provides a 27-item checklist harmonized for both traditional regression and ML-based methods. Alternative frameworks, such as the Minimum Information about Clinical Artificial Intelligence Modeling (MI-CLAIM) [22], may also be applicable depending on the study design. The full TRIPOD-AI checklist is freely available at www.tripod-statement.org (Table 4).

**Table 4:** TRIPOD + AI checklist with six domains. AUC: area under the curve, DCA: decision curve analysis, TRIPOD-AI: Transparent Reporting of a Multivariable Prediction Model for Individual Prognosis or Diagnosis-Artificial Intelligence. Adapted From: [13].

TRIPOD-AI domain	Key items required	Commonly missed?
Title and Abstract	Clearly state it is a prediction model study; include the TRIPOD-AI for Abstract's checklist items (outcome, population, model type, key performance metric)	Yes, vague titles and abstracts that omit AUC or population are common
Introduction	State the specific prediction objective; justify why existing tools are insufficient; define the intended use of the model	Partially, justification is often weak
Methods: data	Describe source, setting, eligibility criteria, outcome definition, predictors, sample size rationale, and how missing data were handled	Yes, missing data handling often omitted
Methods: model	Specify algorithm, hyperparameter tuning approach, train/test split (with random seed), cross-validation method, and threshold used for classification	Yes, random seed and threshold consistently missing
Results: performance	Report discrimination (AUC/C-index) AND calibration AND clinical utility (e.g., DCA). Provide confidence intervals for all metrics	Yes, calibration and DCA almost always absent
Discussion and openness	Discuss limitations including potential bias, external validity, and intended clinical use. State whether code and data are available	Partially, code availability rarely addressed

Part 6: Using AI to write your code

The New Paradigm: AI as Your Coding Partner

In 2026, it is no longer necessary to know Python to build a ML model. What is required is the ability to clearly describe, in plain English, the desired task to an LLM, such as Claude or ChatGPT. The LLM generates the code, which is then executed in Google Colab, while the clinician evaluates the output from a clinical perspective. For standard clinical ML tasks, the code generated by modern LLMs is often correct, well-commented, and consistent with established best practices. The key skill is effective prompting, the ability to precisely define the problem so that the AI produces the desired output within one or two iterations.

Principles of Effective Prompting

Five principles underpin effective AI-assisted coding. First, be specific about your data: clearly describe variable names, data types, and the outcome variable rather than using general terms. Second, state your objective precisely: for example, a prompt such as “Build a Random Forest model to predict BCR (yes/no) using these 12 features, with an 80/20 stratified split and 10-fold cross-validation” will generate more useful output than “Build a Random Forest model.” Third, request explanations alongside code, as asking the AI to explain each section in plain English produces clearer and more interpretable outputs. Fourth, iterate: if the initial output is not correct, describe the issue specifically and request a targeted revision rather than restarting from scratch. Fifth, include error handling by asking the AI to provide informative messages at each step, which makes debugging significantly easier.

Complete Prompt Templates by Pipeline Stage

Loading and exploring your data:

EXAMPLE PROMPT I am a urologist using Google Colab. I have 'my_data.csv' with [N] patients. Columns: [list names and types e.g. 'Age (continuous), PSA_initial (continuous), T_stage_pre (ordinal: T2a/T2c/T3a/T3b/T4), BCR (binary: 0/1)']. My outcome is BCR. Write Python code to: 1) Load file, 2) Show first 5 rows, 3) Count missing values per column, 4) Show the BCR class distribution. Include comments.

Building a logistic regression model:

EXAMPLE PROMPT Using dataframe 'df', outcome 'BCR', features [list], write Python code to: 1) Define X and y, 2) 80/20 stratified split (random_state=42), 3) Scale features with StandardScaler, 4) Train Logistic Regression, 5) Print AUC, sensitivity, specificity, confusion matrix on test set, 6) Plot ROC curve. Explain each section.

Building a Random Forest with feature importance:

EXAMPLE PROMPT Using the same train/test split: 1) Train Random Forest (100 trees), 2) Print test AUC, 3) Plot top 10 feature importances as horizontal bar chart, 4) Run 10-fold CV and report mean ± SD AUC, 5) Save model as 'rf_model.pkl' using joblib.

Cox regression and Kaplan-Meier curves:

EXAMPLE PROMPT I have 'BCR_time_months' and 'BCR_event' (1=occurred, 0=censored) plus predictor variables [list]. Using lifelines: 1) Fit Cox model, 2) Print hazard ratios with 95% CIs, 3) Create forest plot, 4) Divide patients into low/medium/high risk groups, 5) Plot Kaplan-Meier curves with CIs and log-rank pvalue.

Part 7: Building a web application

A prediction model saved as a .pkl file on a local computer has limited clinical utility. Deploying it as an interactive web application transforms it from a static research artifact into a usable clinical tool that can be accessed from any browser, on any device, worldwide. As an illustrative example, a multi-outcome prostate cancer prediction tool was deployed [12] in under 72 hours using entirely free infrastructure, without server management or prior web development experience.

Gradio: Building the Interface

Gradio is an open-source Python library that enables the creation of interactive web interfaces for ML models with minimal code. Users define input variables (e.g., sliders, dropdowns, number inputs) and output formats (e.g., gauges, plots, text), while Gradio handles the underlying web rendering. The resulting interface can be run locally in Google Colab for testing and subsequently deployed to Hugging Face Spaces for public access.

EXAMPLE PROMPT I have a trained model 'bcr_model.pkl'. Inputs: Age (slider 40-90), Initial_PSA (number 0-200), Gleason_Grade_Group (dropdown: 1,2,3,4,5), T_stage (dropdown:T2a/T2c/T3a/T3b/T4), PSA_reduction_pct (slider 0-100). Output: BCR probability (0-1). Write Gradio Python code with: 1) All input controls, 2) Gauge chart showing BCR probability, 3) Text output with risk interpretation (Low/Medium/High), 4) Submit button. Include Colab running instructions.

Deployment platform comparison (Table 5) summarizes six platforms for deploying clinical AI web applications, outlining their cost, required skill level, and key limitations to assist clinicians in selecting the most appropriate hosting option.

**Table 5:** Deployment platform comparison.

Platform	Best for	Cost	Skill level	Limitations
Hugging Face Spaces	ML models, Gradio apps: easiest option for researchers	Free (basic)	Minimal	2GB RAM free; sleeps after inactivity
GitHub Pages	Static sites, documentation	Free	Minimal	Cannot run ML models directly
Render	Robust deployment, never sleeps	Free/$7 per month paid	Low-moderate	Free tier can be slow to start
Railway	Full-stack apps, persistent storage	$5 per month	Moderate	No free tier; requires configuration
Streamlit Community	Python dashboards, data apps	Free	Minimal	Streamlit-specific syntax; less flexible
Google Cloud Run	Production-grade, scalable	Pay-as-you-go	Moderate-high	Requires containerization knowledge

Deploying to Hugging Face Spaces: Step by Step

Deployment to Hugging Face Spaces involves five straightforward steps. First, create a free account at huggingface.co, then click “New Space,” assign a name, and select “Gradio” as the SDK. Next, upload three files: (1) app.py containing the Gradio interface code, (2) a requirements.txt file listing all required Python packages with version numbers, and (3) the trained .pkl model files. Hugging Face will automatically install the specified dependencies and launch the application. Once deployed, the app is accessible at huggingface.co/spaces/[username]/[space-name] from any browser worldwide, without requiring server management.

The requirements.txt file should include all necessary Python packages: for example, gradio, scikit-learn, xgboost, lifelines, pandas, numpy, joblib, and plotly, each pinned to the version used during development.

Part 8: Publishing your work

Match your study to the journal’s scope and impact expectations before writing. Every single-center retrospective AI study should explicitly acknowledge key limitations, including its retrospective design and associated selection bias; single-center data limiting generalizability; absence of external validation; potential overfitting despite cross-validation; use of SMOTE for rare outcomes; and the risk of algorithmic bias if demographic subgroups are underrepresented. The TRIPOD-AI checklist should be completed prior to submission, as commonly omitted items include calibration reporting, specification of the train-test split with a random seed, classification threshold selection, and subgroup performance analysis. Proactively addressing these limitations is essential and may improve the likelihood of manuscript acceptance.

Part 9: Ethics, bias, and responsible AI

Algorithmic Bias in Urological Data

Algorithmic bias occurs when a model systematically performs worse in one patient subgroup compared with another. In urological datasets, the most common sources include racial and ethnic underrepresentation (as most published prostate cancer datasets are predominantly of White race), socioeconomic confounding in tertiary referral centers, and temporal bias when models trained on older datasets are applied to patients managed under current treatment protocols [23]. Even well-validated prostate cancer pathological prediction models have been shown to perform significantly worse in non-White populations during external validation [24]. A practical approach is to evaluate model performance separately across key subgroups and report any disparities transparently and prominently. It is also important to note that patients with localized prostate cancer generally have excellent long-term outcomes when managed with any of the main treatment modalities [25], which should help contextualize the clinical significance and stakes of any prediction tool in this setting.

IRB Approval and Ethical Considerations

Any study using patient data, whether retrospective or de-identified, requires Institutional Review Board (IRB) approval or clearance from an equivalent ethics committee prior to use for research. In many jurisdictions, clinical decision support tools may require additional regulatory review and can be classified as medical devices [26]. The regulation of predictive analytics in medicine is an evolving field, and clinicians deploying AI tools have a professional and ethical responsibility to remain informed of the applicable regulatory requirements.

## Discussion

This guide outlines a structured, nine-part framework designed to help urologists with no prior programming experience develop, validate, and deploy AI-based clinical prediction tools using their own institutional data. Each stage of the process, from data appraisal to web application deployment, is explained in clinically accessible language, supported by practical prompt templates for AI-assisted coding and reinforced with real-world urological examples. A key takeaway is that the technical barrier to AI model development has significantly decreased, largely due to the availability of free, browser-based coding platforms and LLMs that can generate functional code from plain-English instructions.

One of the main strengths of this framework is its relevance to the type of data most urologists already have. Single-center retrospective datasets of around 150-300 patients, common in departmental audits and surgical logs, are sufficient for initial model development, as supported by several published urological AI studies [14-16]. The approach does not depend on advanced research infrastructure, dedicated data science teams, or substantial funding, making it practical for both academic and community-based clinicians.

This framework also addresses a clear gap in current AI education resources. Many existing methodological guides are written for technically trained audiences and assume familiarity with programming and ML concepts that most clinicians do not have [8]. In contrast, this guide is written from a clinical perspective, focusing on how urologists think and approach problems. The use of prompt-based coding positions LLMs as collaborative tools, aligning naturally with how clinicians describe clinical questions. This approach has already shown promise in several medical computing applications.

However, there are important limitations to consider. Models developed using this framework are typically single-center and retrospective, which limits their generalizability. Like all AI prediction models, performance may not translate reliably to different patient populations [11]. As such, this framework should be viewed as an entry point for model development rather than a replacement for prospective, multicenter validation. Additionally, while TRIPOD-AI reporting standards [13] are included, adherence requires careful attention, particularly for elements like calibration, decision curve analysis, and subgroup performance, which are often underreported.

Looking ahead, future work should explore whether consistent use of this framework across multiple centers can produce reliable and clinically meaningful prediction tools, and whether these tools improve patient outcomes compared to standard decision-making. As LLMs and deployment platforms continue to evolve, the technical challenges will likely become less of a barrier. The focus will need to shift toward validation, monitoring, and safe integration into everyday clinical practice.

## Conclusions

AI is no longer a distant concept that urologists can afford to observe from the sidelines. It is already a practical and accessible set of tools that, when applied with clinical rigor and methodological honesty, can extract meaningful insights from existing data, generate validated prediction models, and ultimately improve patient care. The technical barrier to entry has never been lower. The nine-part framework presented here, from data appraisal to ethics and responsible deployment, offers a structured, replicable, and teachable pipeline that can be applied to virtually any structured urological dataset. It is designed to meet clinicians where they are, using the data they already possess and the clinical questions that matter most in their daily practice. We encourage clinicians to start with what they have: their own data, their own patient populations, and the outcomes they care about most. The tools are ready. The methodology is in place. What remains is the decision to use them.
